# Late or Lack of Vaccination Linked to Importation of Yellow Fever from Angola to China

**DOI:** 10.3201/eid2407.171868

**Published:** 2018-07

**Authors:** Rui Song, Shengcan Guan, Shui Shan Lee, Zhihai Chen, Chen Chen, Lifen Han, Yanli Xu, Ang Li, Hui Zeng, Hanhui Ye, Fujie Zhang

**Affiliations:** Beijing Ditan Hospital, Capital Medical University, Beijing, China (R. Song, Z. Chen, Y. Xu, F. Zhang);; Mengchao Hepatobiliary Hospital of Fujian Medical University, Fuzhou, China (S. Guan, L. Han, H. Ye);; The Chinese University of Hong Kong, Shatin, Hong Kong, China (S.S. Lee);; Beijing Key Laboratory of Emerging and Reemerging Infectious Diseases, Beijing (C. Chen, A. Li, H. Zeng)

**Keywords:** yellow fever, viruses, outbreak, vaccination, viscerotropic, jaundice, vomiting, hemorrhage, oliguria, liver enzymes, bilirubin, bleeding, kidney, IL6, nucleic acid, China, Angola

## Abstract

During March and April 2016, 11 yellow fever cases were identified in China. We report epidemic and viral information for 10 of these patients, 6 of whom had been vaccinated before travel. Phylogenetic analyses suggest these viruses nested within the diversity of strains endemic to Angola, where an outbreak began in 2015.

In December 2015, the first case of a major yellow fever outbreak was reported in Angola; the outbreak spread to Democratic Republic of the Congo (DRC) (http://www.who.int/csr/don/13-april-2016-yellow-fever-angola/en/). In addition to 965 confirmed cases in DRC during December 2016–February 2017 (https://reliefweb.int/report/democratic-republic-congo/yellow-fever-outbreak-angola-and-democratic-republic-congo-ends), the outbreak led to exportation to other countries. In China, 11 imported cases were reported during March and April 2016 (*1*); 1 case-patient subsequently died (*2*). We conducted a clinical-epidemiologic study on 10 of the 11 case-patients. The study was approved by the Review Board of Beijing Ditan Hospital (Beijing, China) and the Ethic Committee of State Key Laboratory of Pathogen and Biosecurity. We also obtained informed consent from recruited patients. 

We confirmed the yellow fever diagnoses according to criteria established by the China National Health and Family Planning Commission, in agreement with the recommendations of the World Health Organization (*3*). All case-patients were citizens of China who had stayed in Angola for a period of 5 months to 7 years before returning to China; 7 (70%) were male, the median age was 41 (range 17–50) years (Technical Appendix Table 1). Six case-patients reported a history of yellow fever vaccination; 4 of those were vaccinated <­14 days before symptom onset. Case-patient 7 received vaccination in China 5 years before symptoms occurred, and case-patient 10 was vaccinated in Namibia 10 months before onset of illness. None had received fractional doses of yellow fever vaccine as advocated in some studies (*4*). 

We tested blood samples from all case-patients for yellow fever–specific antibodies by ELISA. We also extracted RNA from blood and urine samples by using the QIAamp MinElute Virus Spin Kit (QIAGEN, Valencia, CA, USA) for reverse transcription PCR virus detection. Phylogenetic analysis (Figure, panel A) of samples from 9 case-patients showed that the sequences were very closely related to each other and resembled the wild-type strain implicated in the Angola outbreak (*2*). We evaluated virus sequences of 5 of the 6 vaccinated case-patients; all showed the same wild-type strain. For the remaining patient (case-patient 7), RNA extraction was unsuccessful. She had been vaccinated 5 years before her illness, excluding the possibility of a vaccine-related adverse event.

**Figure:**
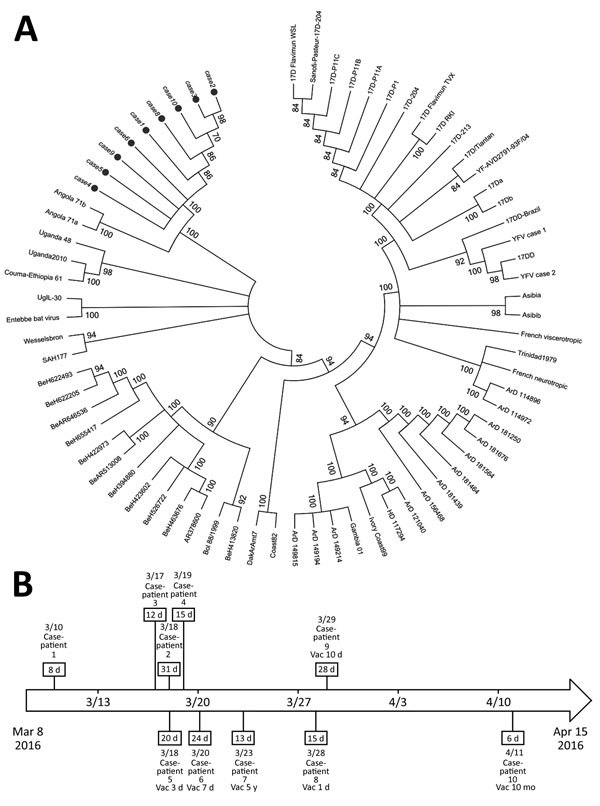
Phylogenetic analysis of yellow fever viruses and clinical courses for persons with yellow fever cases imported from Angola to China. A) Phylogenetic relationships among the yellow fever viruses from samples obtained from 9 case-patients (black circles). An unrooted dendrogram with maximum-likelihood by genome sequences represents the phylogenetic relationships. Clusters with bootstrap support values <70 were integrated; bootstrap values are shown on the branches. B) Dates of hospital admission, detection of virus in urine, and vaccination status for the 10 case-patients investigated. Case-patients 1–4 were not vaccinated. Numbers in boxes indicate longest interval from symptom onset to urine virus sequence detection. Vac, interval from previous vaccination to symptom onset.

Clinically, all 10 case-patients had acute onset of fever lasting 1–7 days; the highest temperature was 39.5°C (Technical Appendix Table 2). The most common symptoms were fatigue, headache, dizziness, and myalgia. Of 10 case-patients, 8 had been treated for malaria before yellow fever was confirmed. Eight case-patients, including 2 of the 4 unvaccinated case-patients, had relatively mild symptoms. Case-patients 1 and 2, both unvaccinated, had severe disease; signs and symptoms were jaundice, vomiting, hemorrhaging (petechiae, ecchymosis, and gastrointestinal bleeding), and oliguria, as well as high levels of liver enzymes (alanine aminotransferase 11,425 and 3,710 µ/L, respectively) and total bilirubin (>100 µmol/L). We also noted bleeding tendency, reflected by a high international normalized ratio and thrombocytopenia. Case-patient 1 deteriorated rapidly as a result of severe kidney and liver damage; biopsy showed evidence of panlobular and confluent hepatocyte necrosis (*2*). His CD4 T cell count was low at 155 cells/µL, but we excluded HIV co-infection. Despite continuous hemofiltration and hemodialysis and mechanical ventilation support, he died 9 days after symptom onset. The remaining 9 patients recovered after hospitalization, which lasted for a median of 16 (range 11–52) days.

Case-patients 1 and 2 also showed strong inflammatory responses as reflected by high plasma level of interleukin 6. Yellow fever virus nucleic acid continued to be detectable in urine during week 1 after symptom onset; case-patient 2 had the longest interval of detection at 31 days (Figure, panel B). 

Overall, the clinical courses of these imported yellow fever cases in China were similar to others reported in the published literature: mild diseases in most case-patients, but high fatality rates among severe cases (*5*). China is not a yellow fever–endemic area, but importation of the virus was not surprising, considering the high number of travelers between Angola and China (*6*). Unfortunately, vaccination coverage was not high for Chinese travelers bound for Angola; among this cohort, only 2 had been vaccinated before travel to a yellow fever–endemic area. Travelers may be worried about vaccine-associated viscerotropic disease (*7*), but this condition did not occur among the vaccinated case-patients in our study, who all had a moderate disease, requiring hospitalization of 11–29 days. The World Health Organization had recommended vaccination at least 10 days before entering a yellow fever–endemic area, but late vaccination in travelers is common (*8*). 

Our observations highlight the importance of timely immunization to achieve protection during an outbreak within a yellow fever–endemic area. Vaccination efficacy and long-term protection are other concerns highlighted in this study: 2 patients were infected despite vaccination received 1–5 years previously. Although most international guidelines did not recommend booster administration of vaccine, its possible role in outbreak settings demands further research (*9*,*10*).

Technical Appendix Characteristics of case-patients with yellow fever imported to China from Angola, 2016.
