# Supplementary material for: Late or Lack of Vaccination Linked to Importation of Yellow Fever from Angola to China
Source: Emerg Infect Dis. 2018 Jul;24(7):1383–6. doi: 10.3201/eid2407.171868 (PMC6038747; doi:10.3201/eid2407.171868)
Supplement: Technical Appendix — Characteristics of case-patients with yellow fever imported to China from Angola, 2016. [file 17-1868-Techapp-s1.pdf]

Article DOI: https://doi.org/10.3201/eid2406.171612

Late or Lack of Vaccination after Angola Outbreak Linked to Yellow Fever, China

Technical Appendix

Technical Appendix Table 1. General characteristics of the case-patients in China who had confirmed diagnoses of yellow fever after being in Angola during the 2015–2017 outbreak (n = 10)

Characteristic		No. median	Range (%)
Male gender		7	(70)
Age (years)		41	(17–50)
Duration of stay in Angola	<1 y	4	(40)
	1–3 y	1	(10)
	3–5 y	2	(20)
	>5 y	3	(30)
Place of diagnosis	Beijing	5	(50)
	Fuzhou	5	(50)
Month of symptom onset	March 2016	9	(90)
	April 2016	1	(100)
History of yellow fever vaccination		6	(60)
Days of hospitalization		16	(11–52)
Deceased		1	(6)

Technical Appendix Table 2. Clinical and epidemiologic characteristics of yellow fever cases imported to China from Angola in March and April 2016 (n = 10)

	Case-patient No.									
Characteristic	1	2	3	4	5	6	7	8	9	10
A) Epidemiologic history										
Date of arrival in China	3/9	3/18	3/17	3/19	3/11	3/19	3/21	3/21	3/27	4/11
Date of symptom onset	3/8	3/11	3/9	3/6	3/10	3/17	3/18	3/13	3/12	4/15
Date of hospital admission	3/10	3/18	3/17	3/19	3/18	3/20	3/23	3/28	3/29	4/11
Date of confirmed diagnosis	3/16	3/18	3/18	3/20	3/24	3/24	3/25	3/30	3/2	4/13
Previous vaccination	No	No	No	No	Yes	Yes	Yes	Yes	Yes	Yes
Interval from vaccination to symptom onset	NA	NA	NA	NA	3 d	7 d	5 y	1 d	10 d	10 mo
B) Clinical signs and symptoms										
Fever	+	+	+	+	+	+	+	+	+	+
Highest temperature recorded C°	39.3	39.1	38.5	38	ND	39	37.3	39.3	39	39.5
Days of fever	1	7	7	3	1	6	1	2	5	3
Hemorrhage	+	+	ND	ND	ND	ND	ND	ND	ND	ND
Oliguria	+	+	ND	ND	ND	ND	ND	ND	ND	ND
Other symptoms	H, D, M, J, V, F	D, M, J, V, F	D, M, F	H, D, M, F	H, M, F	H, D, M, V, F	F	H, D, M, F	H, D, M, F	H, D, M, F
Shock	+	ND	ND	ND	ND	ND	ND	ND	ND	ND
Days in hospital	6	52	28	26	15	27	17	12	11	29
Outcome	Died on day 9	Rec	Rec	Rec	Rec	Rec	Rec	Rec	Rec	Rec

Characteristic	Case-patient No.									
	1	2	3	4	5	6	7	8	9	10
C) Laboratory findings at diagnosis										
Hemoglobin (g/dL)	16.1	17.0	13.7	15.3	13.1	13.9	12.9	11.8	14.1	152
Leukocyte ($\times10^9$/L)	6.23	3.01	3.91	6.06	5.51	2.1	9.41	5.59	6.54	3.07
Platelet count ($\times10^9$/L)	70	70	90	302	204	65	295	254	373	60
ALT (U/L)	11425	3710	323.3	312.8	81	805	139	44	259	1418.1
AST (U/L)	21467	6460	158.8	101.6	49	857	75	40	61	714.1
Total bilirubin (umol/L)	100.6	166.2	8.3	12.2	5.4	8.9	4.7	13	15	14.8
GGT (U/L)	ND	331.5	294	248.7	49	43	139	20	177	631.6
CK (U/L)	670.2	470.4	167.3	58.7	83	140	405	177	176	162.2
Creatinine (μmol/L)	650.1	89	67.2	65.6	56	79	61	60	71	78
BUN (mmol/L)	19.33	6.18	4.13	2.72	2.6	4.2	3.4	7.3	4.6	5.56
IL-6 (pg/ml)	182.6	137.2	<1.5	<1.5	2.79	12.12	10.68	5.77	3.4	ND
CD4(cells/ul)	155	333	1163	717	648	ND	ND	467	1500	798
D) Urine virus sequence detection										
Days from symptom onset	8	31	12	15	20	24	13	15	28	6
Result (CT value)	33.5	25.22	30.95	35.22	39.40	39.39	U	37.06	37.29	23.77

NA, not applicable; ND, no data; +, U, undetectable; H, headache; D: dizziness; M: myalgia; J: jaundice; V: vomiting; F: fatigue.
